# Discriminative and Affective Processing of Touch: Associations with Severity of Skin-picking

**DOI:** 10.1007/s10919-022-00415-4

**Published:** 2022-08-30

**Authors:** Anne Schienle, Albert Wabnegger

**Affiliations:** Department of Clinical Psychology, University of Graz, BioTechMed, Universitätsplatz 2/DG, 8010 Graz, Austria

**Keywords:** Skin picking;, Skin sensitivity, Two-point discrimination, Discrimination of surface texture, Affective touch processing

## Abstract

Skin-picking is a common behavior in the general population that generally serves emotion regulation (e.g., reduction of tension). However, recent research suggests it may also be associated with changes in tactile processing sensitivity. Along these lines, the present study examined whether the severity of skin-picking (SOSP) is related to discriminative and affective touch processing. A total of 160 participants (59 males, 101 females, mean age = 31 years) completed two tactile discrimination tests (two-point discrimination, surface texture discrimination), as well as a well-validated affective touch paradigm (delivery of soft/slow touch, which is found to be generally pleasant). A hierarchical regression analysis was carried out to investigate the association between SOSP, age, sex, and indicators of tactile sensitivity. Replicating previous findings, females reported higher SOSP. While the performance in the discrimination tests did not predict SOSP, affective touch processing was associated with SOSP. Participants with high SOSP reported an urge to pick their skin after being softly touched. This seems paradoxical since previous findings have suggested skin-picking may be carried out to manage negative affective states. Our findings add to the literature describing altered sensitivity and responsivity to specific tactile stimuli in individuals with excessive skin-picking.

## Introduction

Skin-picking is a common behavior in the general population (Bohne et al., 2002; Odlaug et al., 2013). We all occasionally pick at scabs or bumps, pop a pimple, or pick on the cuticles around our fingernails. Evolutionary theories have suggested that skin-picking can be understood as self-grooming, with the aim of cleaning and maintaining parts of the body. Originally, this type of behavior may have served to extract foreign objects and parasites from the skin (Bohne et al., 2002; Prokop et al., 2014). Skin-picking could therefore be seen as a form of hygiene.

Moreover, skin-picking has been associated with emotional functioning. It becomes more frequent in times of emotional distress. Negative feelings (e.g., frustration, anger, boredom) are typical triggers of skin-picking, which helps to reduce tension and is experienced as soothing (Bohne et al., 2002; Gallinat et al., 2021). In this sense, skin-picking can be seen as part of an adaptive effort to manage negative affective states (Neff et al., 2011; Snorrason et al., 2010). Thus, occasional skin-picking may be an adaptive behavior serving emotion regulation.

However, skin-picking can become so frequent and intense that it causes lesions, bleeding, infections, and scars. In that case, it has developed into a mental disorder, labeled as skin-picking disorder (SPD), or excoriation disorder (American Psychiatric Association, 2013). The estimated prevalence of SPD in the general population is 2–3%, while 10–14% show subclinical forms of skin-picking (Grant & Chamberlain, 2020; Machado et al., 2018). People with SPD will often scratch their skin for several hours a day. They may pick at minor skin irregularities (e.g., calluses, pimples), lesions, or scabs, but also healthy skin. Typical target regions for skin-picking are fingers, hands, arms, legs, and the face. Most patients with SPD pick at multiple target regions (Grant et al., 2012). The resulting changes in skin appearance (e.g., open wounds, scars) cause clinically significant distress and/or impairment in important areas of functioning (e.g., people with SPD may feel embarrassed to show their skin openly and may spend a lot of time covering up sores before work or social events, or often avoid these entirely).

Skin-picking typically starts with the inspection of the skin, either visually or in a tactile manner. Regarding the latter, areas of the skin are usually touched systematically, to detect irregularities (e.g., bumps). Fingertips are used to carry out the checking, while the picking is done with the fingernails. This excessive touching of the skin has been linked to sensory over-responsivity (SOR; Falkenstein et al., 2018), which is related to the broader model of sensory processing sensitivity (SPS; Aron et al., 2012). This model holds that many interindividual differences in personality (behaviors, cognitions, and emotional patterns) emerge as a result of an individual being more or less sensitive, responsive/reactive, or flexible to sensory stimuli in the environment (Aron et al., 2012). Findings regarding differences in sensitivity to certain sensory stimuli in those affected by skin-picking are discussed in the following.

Two recent studies (Falkenstein et al., 2018; Houghton et al., 2018) have shown that individuals who execute body-focused repetitive behaviors (BFRBs: hair-pulling, skin-picking) tend to display heightened reactions to ordinary sensory stimuli, such as clothing textures (e.g., being bothered when touching soft towels, or wearing a turtleneck). In a study by Falkenstein et al. (2018), participants with trichotillomania (pathological hair-pulling) reported higher auditory and tactile responsivity than comparison participants. Houghton et al. (2018) found that individuals with clinical BFRBs (skin-picking, cheek biting, hair-pulling) showed greater sensory sensitivity (and sensory avoidance) than individuals with subclinical BFRBs and healthy controls. In a further study (Houghton et al., 2019), individuals with pathological hair-pulling and skin-picking exhibited increased touch sensitivity, as reflected by decreased detection thresholds for a vibratory stimulus. In sum, this research suggests differences in sensitivity to sensory stimuli in individuals with BFRBs such as skin-picking.

In the current study, the association between skin-picking and the processing of touch was investigated. Generally, touch has discriminative and affective properties (McGlone et al., 2014). Firstly, it is important for humans to rapidly detect, discriminate, and identify external stimuli that touch the skin (e.g., a sharp object that touches the hand). This allows us to make rapid decisions that guide subsequent behavior (e.g., pulling back of the hand). Discriminative touch information is transmitted via rapidly conducting large myelinated fibers that project to cortical regions such as the somatosensory cortex. The second type of touch information, affective touch, is mediated by C-tactile (CT) afferents (slow, unmyelinated fibers) to somatosensory, and additionally to limbic regions concerned with affective processing (McGlone et al., 2014). These types of mechanoreceptors are activated by soft touch (caressing).

A well-validated paradigm in experimental studies for affective touch consists of administering slow and soft brush strokes to the skin of the participants (McGlone et al., 2014). It has been shown that brushing velocities between 1 and 10 cm/s are optimal for activating CT afferents (Ackerly et al., 2014). This activation is correlated with reported feelings of pleasantness (Löken et al., 2009). In the first study on affective self-touch in patients with SPD, Schienle et al. (2018) observed, relative to healthy controls, reduced sensitivity for slow stroking (caressing) in those with SPD.

In the present study, we used the affective touch paradigm described above (McGlone et al., 2014). Participants received slow (CT-optimal: 3 cm/s) brush stroking to their forearms by an experimenter, as well as fast brush stroking (30 cm/s) as the control condition. In adults, interpersonal affective touch has been associated with the induction of positive feelings (pleasure), and also with the reduction of subjective as well as somatic components of emotional distress and pain (for a review, see Saarinen et al., 2021). The stress-buffer hypothesis of affective touch holds that being gently stroked by another person can positively influence emotion regulation (Morrison, 2016).

In addition to assessing affective touch, the current study also investigated indicators of discriminative touch performance (two-point discrimination at the tip of the dominant index finger, and discrimination of surface textures via the index finger). A hierarchical regression analysis was carried out to investigate possible associations between the severity of skin-picking (SOSP) and tactile processing (discriminative, affective) in a large non-clinical sample.

## Method

### Participants

A total of 160 participants (59 males, 101 females, mean age = 31.42 years, SD = 13.37; 93% right-handed) were recruited at a university and via social media postings. They were invited to a study on touch processing. The majority of participants had a high school diploma (84%). Forty percent were university students; the remaining participants were white-collar workers (56%) or retired (4%). Exclusion criteria were reported somatic conditions that affect touch processing (e.g., neurological disorders) and dermatological conditions (e.g., psoriasis).

All participants provided informed consent. The study was preregistered on the open science framework (https://osf.io/uytp9/) and approved by the local ethics committee. The study was supported by the Austrian Science Fund (KLI 824).

### Measures

The participants answered the German version of the Skin Picking Scale-Revised (Gallinat et al., 2016), which assesses symptom severity and impairment due to skin-picking during the last week. The eight items (e.g., How strong was your urge to pick your skin?) are answered on 5-point scales (0 = *no urge*; 4 = v*ery strong urge*). An overall score (total SPS_R; Cronbach’s alpha = 0.90) was computed that reflects the severity of skin-picking (SOSP).

Moreover, the participants completed three tactile tasks in randomized order while being blindfolded.

#### Two-Point Discrimination

The W54670 (baseline) sensitivity tester (Fabrication Enterprises Inc.; model number: 12–1492) was used to assess the ability of the participants to discern two nearby points at the tip of their dominant index finger (two-point distances ranging between 2 and 5 mm; control stimulus: one point). The test used a forced-choice technique; the participants were prompted to select one of the following options: ‘one point’ or ‘two points’. The assessment followed a staircase method of descents and ascents. The two-point threshold was defined as the smallest distance at which 7 out of 10 two-point stimuli were correctly identified.

#### Discrimination of Surface Textures

Seven different grades of sandpaper, each with a different level of coarseness (P120, 180, 240, 320, 600, 800, 1000) were presented to the participants in pairs (size of each paper: 23 cm × 9.2 cm). Coarseness (grit size) refers to the size of the particles of the abrading material embedded in the sandpaper (higher values indicate less coarseness). The participants were asked to move their index finger (of the dominant hand) downwards over each surface, and decide whether the coarseness of the two papers was different or not (forced-choice). The differentiation sensitivity index (determined via a staircase method) reflects that 7 out of 10 pairs of the same grade were correctly identified.

#### Affective Touch Evaluation

A well-validated paradigm was used to administer CT-optimal (3 cm/s) and suboptimal (30 cm/s) brush stroking to the left forearm of the participants (for a review see Cruciani et al., 2021). The stroking was delivered by a trained experimenter (stroking distance: 6 cm) in a proximal to distal direction. Both types of stroking (slow, fast) were administered for 30 s each in a balanced order. The stroking speed applied was controlled by a metronome via headphones. After 30 s of each stroking condition, the participants rated the touch experience according to pleasantness, arousal, and urge to pick their skin on a 9-point scale (9 = *very pleasant, very aroused, high urge to pick*).

### Statistical Analysis

A hierarchical multiple linear regression analysis (with three blocks) was computed to examine the relationship between SOSP and the predictor variables age, sex (first block), the two scores for discriminative touch performance (two-point/ coarseness discrimination; second block), and the six ratings for the affective touch experience (pleasantness/arousal/urge to pick for slow/fast stroking; third block). The model was assessed for multicollinearity and distribution of residuals. Age and the six ratings for the affective touch experience were mean-centered. For the affective touch test, we computed paired t-tests to compare the two stroking speeds (slow, fast) according to reported pleasure, arousal, and urge to pick one’s skin. All tests were conducted using the Statistical Package for the Social Sciences (SPSS, version 26).

## Results

Descriptive statistics for the questionnaire and the tactile tests are displayed in Table 1. The SPS_R scores were M_severity = 2.84 (*SD* = 3.71), and M_impairment = 0.87 (*SD* = 1.73). Twenty-four percent of the participants (31 females, 8 males) had a total SPS_R score ≥ 7 (cutoff score for pathological skin-picking).

**Table 1:** Means (standard deviations) and Pearson correlations between questionnaires and tests

	*M*(*SD*)	2	3	4	5	6	7	8	9
1. Skin-Picking Scale (revised); total score	3.71 (5.20)	−.20 +	.10	−.10	.10	.37**	−.09	.13	.30**
2. Two-point discrimination (mm)	2.89 (0.93)		−.07	.01	−.08	−.01	.06	−.13	−.13
3. Coarseness discrimination index	598 (185)			−.04	.02	.17 +	−.03	.01	.13
4. Slow touch; pleasantness	7.70 (1.62)				−.17 +	−.47**	.38**	−.08	−.12
5. Slow touch; arousal	2.29 (1.68)					.15	−.17 +	.56**	.20 +
6. Slow touch; urge to pick	1.46 (1.44)						−.13	.09	.38**
7. Fast touch; pleasantness	5.34 (2.04)							−.22*	−.30**
8. Fast touch; arousal	2.59 (1.94)								.28*
9. Fast touch; urge to pick	1.94 (1.83)								

+ *p* < .05, *p < .01, **p < .001

Age correlated negatively with the total SPS_R score (*r* = −0.29, *p* < 0.001). Females had a higher total SPS_R score (*M* = 4.70, *SD* = 5.90) than males (*M* = 2.00, *SD* = 3.68, *t* (158) = 3.81, *p* < 0.001).

The paired t-tests showed that slow stroking was associated with more pleasure, *t*(159) = 14.40, *p* < 0.001, less arousal, *t*(159) = −2.22, *p* = 0.028, and less urge to pick one’s skin, *t*(159) = −3.30, *p* = 0.001, than fast stroking.

The overall model for the hierarchical regression analysis including all variables accounted for 28% of the variance, *F*(10,149) = 5.67, *p* < 0.001. Age, sex, and urge to pick one’s skin during slow stroking were predictors of severity of skin-picking (SOSP) (Table 2). The performance in the discriminative touch tests was not associated with SOSP. The control variables in block 1 accounted for 15% of the variance in SOSP. The discriminative test scores entered in block 2 accounted for an additional 2% of the variance. Adding the ratings for the affective touch processing in block 3 increased explained variance by 11%.

**Table 2:** Results of the hierarchical regression analysis

Variable	*R*^2^	beta	*SE*	Confidence interval (lower – upper)	*t*	*p*
Step 1	.15					
Intercept		**2.04**	**.42**	**1.21–2.89**	**5.01**	** < .001**
Sex		**2.64**	**.71**	**1.30–4.13**	**3.33**	**.001**
Age		−**.11**	**.03**	−**.17–**−**.06**	−**3.90**	** < .001**
Step 2	.17					
Intercept		1.91	2.46	−3.2–6.33	.77	.440
Sex		**2.53**	**.70**	**1.22–4.02**	**3.20**	**.002**
Age		−**.10**	**.03**	−**.17–.05**	−**3.49**	** < .001**
Two-point discrimination		−.66	.39	−1.40–.15	−1.55	.123
Coarseness discrimination		.51	.45	−.32–1.46	1.06	.293
Step 3	.28					
Intercept		3.94	2.47	−1.18–8.6	1.64	.102
Sex		**1.85**	**.70**	**.46–3.30**	**2.36**	**.019**
Age		−**.09**	**.03**	−**1.54–**−**.04**	−**3.08**	**.002**
Two−point discrimination		−.63	.43	−1.43–.28	−1.54	.128
Coarseness discrimination		.10	.43	−.70–1.01	0.21	.833
Slow touch; pleasantness		.21	.32	−.42–.89	0.76	.447
Slow touch; arousal		−.04	.29	−.57–.57	−0.15	.877
Slow touch; urge to pick		**1.1**	**.36**	**.40**–**1.85**	**3.43**	** < .001**
Fast touch; pleasantness		.00	.28	−.60–.46	−0.00	.999
Fast touch; arousal		.09	.24	−.41–.55	0.40	.690
Fast touch; urge to pick		.32	.32	−.32–.92	1.35	.180

Bold values indicate *p* < .05

Reference level sex = female; slow touch: stroking 3 cm/s; fast touchh: stroking 30 cm/s; confidence intervals are bootstrapped (*n* = 1000; bias corrected and accelerated)

## Discussion

The current study examined the relationship between the severity of skin picking (SOSP) and discriminative and affective aspects of touch processing. Results showed that the performance in tactile discrimination was not associated with SOSP. Neither the two-point discrimination threshold nor the ability to discriminate surface-texture predicted participants’ scores on the revised version of the skin-picking scale. These results were unexpected. Routines involved in skin-picking consist of repeated skin touching to detect small irregularities (e.g., small bumps, scratches); individuals with subclinical SPD and patients with a clinical diagnosis often engage in these time-consuming touching rituals, searching for skin imperfections. This practice was expected to be associated with improved tactile discrimination sensitivity, which was not the case in the present investigation. However, it should be noted that the discriminative tactile sensitivity in humans is generally very high. Surface textures with average particle sizes in the range of μm (one micrometer) can usually be reliably detected (Miyaoka, 1999).

In contrast, responsivity to affective touch was found to be associated with SOSP. Those participants who reported higher SOSP felt a greater urge to pick their skin after being gently touched. This is a surprising finding. Being gently touched by another person is usually perceived as pleasant and soothing (Löken et al., 2009). In this case, skin-picking as a regulation strategy for negative affective states, should not be required. At the same time, it has been shown that when tactile stimulation of low intensity and velocity is perceived as unpleasant, the carrying out of BFRBs may be triggered; this has been demonstrated in studies on sensory over-responsivity in patients with pathological skin-picking (e.g., Falkenstein et al., 2018). Patients with SPD, for instance, have reported that the feeling of specific clothing textures (e.g., drying themselves with a soft towel after a shower) bothered them and they tried to avoid it. Of note, the slow movement of a towel over the skin does have similar sensory properties as the soft/slow brushing of the skin as applied in the current experiment.

Replicating previous findings, we found that sex was a predictor of SOSP. Research suggests that skin-picking and SPD occur much more frequently in women (Odlaug et al., 2013). Moreover, with increasing age, participants reported a lower propensity to pick their skin. Previous research has demonstrated that individuals with clinically relevant BFRBs report that the symptoms of their picking, although waxing and waning in intensity, remain essentially unchanged over time (Grant et al., 2012). However, longitudinal studies with longer observation intervals are still lacking.

The current study has the following limitations. The results are based on a self-selected sample of participants (with a high educational level), who responded to an invitation for a study on touch processing. This strategy for participant recruitment might be associated with the high prevalence of pathological skin-picking (24% above the clinical cut-off) observed in the present sample.

In conclusion, the present findings point to an association between skin-picking behavior and responsivity to specific emotionally relevant tactile stimuli (here, affective touch). In a previous study using brain imaging, it was also found that individuals with high SOSP displayed atypical responses to soft touch. They were not responsive to gentle self-touch and showed pronounced hypoactivation (Schienle et al., 2018). Consequently, in this group, this type of touch may not be able to be used as a tool for emotion regulation. As an alternative, more intense tactile stimulation (skin-picking) may be chosen to fulfill the same function. In line with this hypothesis are clinical observations which indicate that a majority of patients with SPD describe their skin-picking as soothing; a term that is typically used to describe the effects of soft touch (Gallinat et al., 2021). To the best of our knowledge, the role of touch experiences and attitudes toward (soft) touch has not been examined in samples with subclinical/clinical SPD. Indeed, the investigation of such topics surrounding touch and SPD, including touch by friends and family, intimate touch, childhood touch, and attitudes to self-care, might improve our understanding of dysfunctional skin-picking (Trotter et al., 2018). These aspects should be addressed in future studies.
